# Comprehensive Review on the Biocontrol of *Listeria monocytogenes* in Food Products

**DOI:** 10.3390/foods13050734

**Published:** 2024-02-28

**Authors:** Leontina Grigore-Gurgu, Florentina Ionela Bucur, Octavian Augustin Mihalache, Anca Ioana Nicolau

**Affiliations:** 1 Faculty of Food Science and Engineering, Dunarea de Jos University of Galati, 47 Domneasca Street, 800008 Galati, Romania; leontina.gurgu@ugal.ro (L.G.-G.); florentina.bucur@ugal.ro (F.I.B.); augustinoctavian.mihalache@unipr.it (O.A.M.); 2 Department of Food and Drug, University of Parma, Parco Area delle Scienze 17/A, 43124 Parma, Italy

**Keywords:** bacteriophage, lactic acid bacteria, bacteriocins, endolysins, antilisterial, listeriosis, active packaging

## Abstract

*Listeria monocytogenes* is a foodborne pathogen that causes listeriosis, a group of human illnesses that appear more frequently in countries with better-developed food supply systems. This review discusses the efficacy of actual biocontrol methods combined with the main types of food involved in illnesses. Comments on bacteriophages, lactic acid bacteria, bacteriocins, essential oils, and endolysins and derivatives, as main biological antilisterial agents, are made bearing in mind that, using them, food processors can intervene to protect consumers. Both commercially available antilisterial products and solutions presented in scientific papers for mitigating the risk of contamination are emphasized. Potential combinations between different types of antilisterial agents are highlighted for their synergic effects (bacteriocins and essential oils, phages and bacteriocins, lactic acid bacteria with natural or synthetic preservatives, etc.). The possibility to use various antilisterial biological agents in active packaging is also presented to reveal the diversity of means that food processors may adopt to assure the safety of their products. Integrating biocontrol solutions into food processing practices can proactively prevent outbreaks and reduce the occurrences of *L. monocytogenes*-related illnesses.

## 1. Introduction

*Listeria monocytogenes* is a foodborne pathogen responsible for listeriosis, the fifth most reported zoonosis in humans in the European Union (EU). In 2022, in Europe, 2.738 listeriosis cases were reported, with a notification rate of 0.62/100,000 individuals leading to 1.330 hospitalizations (48.6%) and 286 deaths (10.4%). *L. monocytogenes* was also responsible for 35 foodborne outbreaks and 296 foodborne illnesses-related to the outbreaks, with 242 hospitalizations (81.8%) and 28 deaths (9.5%) [1]. Listeriosis mainly affects vulnerable consumer groups such as children, pregnant women, the elderly, and individuals with immunocompromised systems. This is also validated by the fact that most of the foodborne illness cases were reported in the age group of over 64 years old [1]. While in healthy individuals listeriosis manifests itself as mild influenza and gastroenteritis, in vulnerable consumers, it results in severe symptoms like septicemia, meningitis, and miscarriage/stillbirth [2]. *L. monocytogenes* is often associated with pig meat and products thereof, fish and fish products, mixed food, vegetables and juices, and dairy products other than cheese. This is confirmed by the Rapid Alert System for Food and Feed (RASFF), in which 340 alerts were reported in the last three years, mainly with *L. monocytogenes* in fish, meat, and dairy products [1]. *L. monocytogenes* is a food safety threat due to its ubiquitous nature and ease of entering the processing environment through raw ingredients. Some strains of *L. monocytogenes* can survive for many years and serve as a source of ongoing cross-contamination due to their ability to cling to a range of abiotic surfaces [3,4,5]. Out of 37,779 samples tested at the manufacturing stage, 578 (1.53%) were positive for *L. monocytogenes.* In 2022, the highest notification rate and number of cases of listeriosis were recorded since 2007, indicating the need for ongoing research and mitigation strategies for the reduction in *L. monocytogenes* [1]. However, *L. monocytogenes* is known to be a difficult organism to eradicate even when the best safety management plans are implemented [6,7]. 

In this review, we will discuss the efficacy of actual biocontrol methods against *L. monocytogenes*, future challenges, and perspectives for the scientific community, industry, and the organizational bodies responsible for food safety. Hence, the objectives of this review were to identify the most used biological control methods to fight against *L. monocytogenes* based on the type of food, with the aim to provide a clear picture of the factors that the actors involved in the food chain have to take into consideration when deciding what biocontrol to use and to help them be more efficient in designing a successful control strategy against this pathogen. 

## 2. Biological Control of *L. monocytogenes* in Food

Nowadays, biological controls using bacteriophages, competitive bacterial species like lactic acid bacteria (LAB), bacteriocins, essential oils, and endolysins and derivatives are available for the prevention and control of the growth of *L. monocytogenes* in food. 

Bacteriophages are viruses that infect bacteria with high specificity to their hosts, affecting, in general, one species or one genera of bacteria. They are highly adaptable and can evolve to overcome bacterial resistance, a major problem when using antibiotics and other antimicrobials. These facts make them extremely suitable for the biocontrol of undesired microorganisms in foods, while being harmless to consumers [8]. In the United States, the use of bacteriophages as antimicrobial agents against *L. monocytogenes* in RTE meat products and poultry was first approved by the Food and Drug Administration (FDA) in 2006. The European Union and the European Food Safety Authority (EFSA) evaluated bacteriophages for food application and concluded that they can be very effective for the decontamination of foods of animal origin. However, the report also highlighted that there may be some limitations regarding their efficiency in the case of recontamination [9]. Nevertheless, there are several challenges that phage biocontrol has to overcome before being used on a larger scale. These are the technical aspects, such as developing and implementing methods that ensure a full contact between the phages and the target bacteria depending on the specifics of the food matrix, and educating consumers with regard to the advantages of this novel way to increase food safety over the traditional methods [10]. Furthermore, the food physicochemical parameters, the initial concentration of phages and bacteria, the time of storage, and the food type have a significant correlation with how much *Listeria* is reduced. In general, the higher the initial phage/host ratio, the more effective the phage is in reducing bacterial populations. Moreover, the phages inoculated in liquid foods can move almost freely, and thus, their potential contact and distribution with their host cells is not an issue. However, on solid foods, such as hot dogs, salad leaves, and other produce with rough surfaces, and where the surface area or properties are restricted, phages are less effective than in liquid foods. Due to their high specificity on targeted bacteria, they can be used in dairy without the risk of affecting the starter cultures, represented by lactic acid bacteria. 

Endolysins are peptidoglycan hydrolases produced by bacteriophages during the final step of the lytic cycle, namely, the degradation of the host’s cell wall and its lysis to release the newly developed virions. 

Endolysins are known as “enzybiotics”, which emphasize the relation between their structure and function, with a high specificity, low risk to produce resistance, and the ability to act synergistically with other antibacterial agents [11]. The architecture of most endolysins, which are derived from phages with specificity for Gram-positive bacteria, shows two connected domains. These include the C-terminal domain that provides the binding specificity to the receptors from the bacterial cell wall and the N-terminal domain that has the “important mission” to cleave either the glycan part (glycosidases) or the amide bond (amidases) from the glycan and L-alanine [10,11,12]. 

Lately, these enzymes have been regarded as an effective tool for the biocontrol of pathogenic bacteria in food, considering that they are safe for human consumption and do not change the organoleptic and textural properties of the products [13,14]. Furthermore, a few of them highlighted a good enzymatic activity at 4 °C, in matrices with Ca^2+^ and Mg^2+^ ions that can interfere in the substrate binding mechanism (e.g., LysZ5), or the thermal resistance (e.g., Ply511, Ply118, PlyP35), respectively [15,16]. The use of endolysins to prevent the growth of *L. monocytogenes* in food systems is a relatively recent approach. However, scientific studies investigating its effectiveness across various food types remain limited, at least in connection with meat and meat products. However, the only application registered with the FDA regarding an endolysin preparation that has a GRAS status belongs to Nomad (Bioscience GmbH, Halle, Germany). More precisely, the preparation contains six recombinant endolysins with an antimicrobial activity against *Clostridium perfringens*, being applied in levels up to 10 mg/kg of cooked food [17].

In the last years, the use of lactic acid bacteria (LAB) as an alternative method to chemical preservatives has gained an increased interest from researchers and food producers, in the attempt to satisfy the consumers’ demand for healthier and safer food products [18]. The antagonistic effect of LAB against foodborne pathogens is based on the production of antimicrobial metabolites, including bacteriocins, organic acids, diacetyl, H_2_O_2_, and CO_2_ [19]. 

Bacteriocins are antimicrobial peptides produced by lactic acid bacteria (LAB) as a defense mechanism against competing bacteria and pathogens [19,20,21]. So far, three main classes of bacteriocins have been identified and characterized. Among these, class I and class II bacteriocins have been the most utilized antimicrobial compounds in food preservation attempts. Lantibiotics, the bacteriocins belonging to class I, are small peptides (<5 kDa), which contain post-translationally modified residues (lanthionine, β-methyllanthionine, dehydrobutyrine, dehydroalanine) [22]. Some examples of bacteriocins from this family are nisin, lacticin, mutacin, and lactocin [23]. Class II bacteriocins are non-modified antimicrobial peptides with the size of less than 10 kDa, which are characterized by stability to heat. This class comprises four major sub-classes, namely, class IIa, class IIb, class IIc, and class IId. Class IIa bacteriocins are pediocin-like peptides active against *L. monocytogenes*, with representatives like pediocin PA1 produced by *Pediococcus acidilactici*, leucocin A (*Leuconostoc gelidum*), and enterocin A (*Enterococcus faecium*) [24]. Class IIb includes two-peptide bacteriocins, such as lactococcin G and plantaricin A [25]. The third class (class IIc) contains circular bacteriocins. Some of the reported bacteriocins belonging to this class are enterocin AS-48, circularin A, carnocyclin A, and lactocyclicin Q [26]. Class IId bacteriocins are linear non-pediocin like peptides. Some more recently discovered examples are garvicin A, garvicin AG1, and garvicin AG2 [27].

Among the bacteriocins produced by LAB, nisin was the most investigated lantibiotic in terms of *L. monocytogenes* biocontrol in foods. To date, nisin is still the only bacteriocin approved as a food additive (E234), and its quantity safe for regular human consumption is 2.9 mg/person/day [28]. It gained the status of GRAS from the World Health Organization, Food and Drug Administration, and European Food Safety Authority. Its usage is currently implemented in more than 48 countries in a variety of foods, such as meat products, dairy products, and vegetable products [29,30,31,32]. The antimicrobial activity of nisin against *L. monocytogenes* has been proven a long time ago [33]. Also, for using the bacteriocinogenic LAB starter cultures directly in the foods, no regulatory approval is needed [34]. 

Essential oils (EOs) have gained increased attention in terms of food preservation. Among aromatic plants, thyme (*Thymus vulgaris* L.), rosemary (*Rosmarinus officinalis* L.), cinnamon (*Cinnamomum cassia*), oregano (*Origanum vulgare*), and clove (*Syzygium aromaticum*) have been shown to be valuable sources of EOs and bioactive substances with a great inhibitory effect against undesired bacteria in foods [35,36]. EOs have been assigned the GRAS status by the U.S. FDA, and a list of all approved EOs for food applications can be found in the Code of Federal Regulations (CFR), Title 21, Part 182.20 (21 CFR 182.20). Moreover, the European Commission [37] approved in 2008 the list of EO compounds, which is periodically updated, with additives accepted in many types of food products, based on their antimicrobial properties, aroma, flavors, and preservation properties. Because the EO effectiveness is corelated with the type of oil, its concentration, and compatibility (e.g., solubility, stability, interaction with other compounds) with the food matrices, the regulatory agencies evaluated the safety of EOs based on their chemical composition, concentrations, and potential adverse effects, even the health risks. In any case, the food products containing EOs must be correctly and accurately labeled. EOs are hydrophobic aromatic oils produced by plants as secondary metabolites to protect themselves against pests [38]. Due to the antimicrobial properties of their constituents (terpenoids, aldehydes, ketonic bodies, and phenols) and the status of generally being recognized as safe (GRAS), EOs have been widely used as preservatives in food and cosmetic industries [39,40,41]. However, the use of EOs as food ingredients has several limitations. Their intense aroma can alter the organoleptic properties of foods, which may become unacceptable for consumers. Bearing in mind this inconvenience, food processors have to use EOs at low concentrations, which, in turn, due to the possible interaction with food constituents (fats, starch, or proteins) [42] and external factors (light, oxidation, or heating) [43] may result in poor antimicrobial activity or even inefficiency. Therefore, researchers are looking for alternative ways of use so that both consumers’ acceptance and food safety can be achieved. One effective strategy is to apply EOs in the active packaging of food products. This implies the migration of the active compounds from packages to foods, providing protection against microorganisms [43].

## 3. Biocontrol of *L. monocytogenes* in Meat and Meat Products

Over time, meat and meat products, especially the ready-to-eat (RTE) ones, have been reported as being a major food vehicle for *L. monocytogenes* transmission to humans [44,45,46]. The cause of this phenomenon is mainly attributed to the contamination during processing or post-processing steps, such as slicing and packaging, followed by the growth of the pathogen during storage to numbers that endanger the consumers’ health [46,47,48]. Despite the implementation of strict sanitation and disinfection programs in meat processing facilities, *L. monocytogenes* is still able to survive and persist on floors, drains, and equipment as established biofilms, which become a continuous reservoir of contamination [49,50,51]. Thus, new strategies to control this pathogen in meat and meat products are required. In the attempt to satisfy the consumers’ demand with respect to both healthy and safe foods, recent studies have focused on biocontrol methods, including bacteriophages, antagonistic microbial interactions, and plant- or microbe-derived substances having antilisterial activity.

### 3.1. The Use of Bacteriophages in Meat Products

Nowadays, two phage biocontrol products against L. monocytogenes are commercially available: ListShield^TM^ (formerly known as LMP-102^TM^) produced by Intralytix Inc. (Baltimore, MD, USA) and PhageGuard Listex™ (formerly known as ListexTM or P100) produced by Micreos Food Safety (Wageningen, The Netherlands). The LMP-102™ is a mixture of six purified phages with specific activity against the pathogen that could be applied on the surface of the meat products by spraying at a level not exceeding 1 mL per 500 cm^2^ [52]. Unlike the ListShield phage product, the PhageGuard Listex contains only one phage, P100 [53].

Table 1 summarizes studies regarding the efficacy of commercial antilisterial bacteriophages aimed to control *L. monocytogenes* in meat and meat products. The degree of *L. monocytogenes* reduction has been shown to depend on several factors: the ratio between bacteriophages titer and contamination level [54], diversity of pathogenic strains [54], the contact between the phages and the host [55], occurrence of host resistance to phages, products’ chemical composition and characteristics, and storage conditions [54].

Regarding the contact between bacteriophages and *L. monocytogenes* cells contaminating the meat products, one study tested the efficiency of *Listeria* bacteriophage A511 in a cooked-meat model system under multiple scenarios: both bacteriophage and pathogen in the meat, bacteriophage in the meat and pathogen on its surface, pathogen in the meat and bacteriophage on its surface, and both bacteriophage and pathogen on the meat surface. The research revealed that the phages’ ability to control the growth of *L. monocytogenes* on the meat product is limited because their direct contact with the targeted bacterial cells is limited [55].

### 3.2. Endolysins in Meat Products

There are no sufficient data available in the scientific literature regarding the use of endolysins to control *L. monocytogenes* in meat and meat products. The inactivation of *L. monocytogenes* by endolysins in combination with high pressure processing (HPP) was described by Nassau et al. [62]. Three strains of *L. monocytogenes* (ATCC 15313, WSLC 11043, WSLC 11048) were co-incubated with different endolysin concentrations, ranging from 0.16 mg/mL to 20 mg/mL for PlyP40 and Ply511, respectively, and 100 mg/mL for PlyP825. The enzyme activity was assessed at 90 and 180 min prior to HPP treatments. The HPP parameter level of 200 MPa maintained for 2 min is usually too low to kill the pathogenic cells, when this type of treatment is applied without any other cell sensibilization. Interestingly, the results obtained highlighted a good reduction in *Listeria* spp. cells (up to 5 log CFU/mL), when a synergic effect between endolysins and the HPP treatment (200 MPa/2 min/30 °C) was obtained. The use of endolysins not only significantly enhanced the bactericidal impact of HPP but also facilitated the deactivation of bacterial cells at considerably lower pressure thresholds [62]. A similar strategy by combining endolysin PlyP825 and HHP processing was applied to inactivate *L. monocytogenes* artificially inoculated in smoked salmon, in a concentration of 10^7^ CFU/g. The results showed a reduction of only 1.6 log cycles even when a higher level of endolysin (34 µg/mL) and HPP treatment (500 MPa/10 min/25 °C) were used [63].

### 3.3. Lactic Acid Bacteria (LAB) in Meat Products

Another biocontrol method used to prevent the proliferation of *L. monocytogenes* in meat products is fermentation either occurring naturally, as a result of indigenous LAB presence, or stimulated by adding starter cultures. Fermentation results in pH decrease by the formation of lactic acid. Following fermentation, meat products need to be subjected to a drying step, so that the final water activity drops below the limit that allows *L. monocytogenes* to grow. On the other hand, the inhibitory effect of LAB against the pathogen during fermentation may be caused by the production of antimicrobial peptides called bacteriocins. 

Several studies evaluated the behavior of *L. monocytogenes* in fermented meat products in terms of interaction between the pathogen and LAB. Huang et al. [59] showed that LAB addition at a concentration of ~7 log CFU/g to meat sausages subjected to simultaneous fermentation and drying (incubation at 30 °C and relative humidity RH of 76% for 5 days) caused the inhibition of the *L. monocytogenes* population (initially inoculated at ~5 log CFU/g) growth. Moreover, the number of pathogenic cells indicated a slow decrease during the process, by ~0.5 log CFU/g. A similar experiment was reported by Giello and colleagues [64] who co-cultured *L. monocytogenes* OH and Scott A (10^4^ CFU/g) and *Lactobacillus curvatus* 54M16 (10^7^ CFU/g), a strain producing bacteriocins, in sausages ripened for three days at 20 °C (RH: 75–85%) followed by other 25 days at 15 °C (RH: 65–70%). Their results showed that the number of *L. monocytogenes* decreased under the detection limit within the 5 days of co-incubation at 15 °C. Moreover, after 48 h at 15 °C, the only surviving strain was the OH strain, as this was demonstrated by the RAPD-PCR profile [64]. 

The effect of the product’s changing pH on *L. monocytogenes* capacity to multiply during fermentation was also assessed. Kamiloğlu and co-workers [65] concluded that the reduction in *L. monocytogenes* population (2.74 log CFU/g) during the ripening of sucuk (Turkish sausages), for 11 days, was especially due to the fast acidification (pH below 5) caused by the autochthonous *L. plantarum* S50, added as starter culture. The authors did not exclude the antagonistic activity of LAB against the pathogen as a supplementary inhibitory factor, as the strain was confirmed to produce bacteriocins by in vitro tests [65]. 

An innovative approach to benefit from LAB biopreservation potential is to incorporate postbiotics, namely, the substances released during their growth, such as bacteriocins, organic acids, carbon dioxide, and di-acetylene, into polymeric films, which are then used as active packaging materials. In this regard, Beristain-Bauza and co-workers [66] supplemented whey protein films with *L. sakei* cell-free supernatant and used them to wrap beef cubes artificially contaminated with *L. monocytogenes* (~3 log CFU/g). The antimicrobial film reduced *L. monocytogenes* population by 1.4 log CFU/g during refrigerated storage (4 °C) for 120 h [66]. More recently, Shafipour et al. [67] obtained an antimicrobial meat wrapping paper based on bacterial nanocellulose that contained postbiotics produced by *L. plantarum*. The nanopaper proved to have a strong antilisterial activity, as it was shown to reduce *L. monocytogenes* counts in ground meat by ~5 log CFU/g after storage at 4 °C for 9 days [67].

### 3.4. Bacteriocins in Meat Products

Nisin’s efficacy in reducing *Listeria* spp. cells in meat and meat products has been demonstrated in various studies [68,69,70]. However, the occurrence of nisin resistance in *L. monocytogenes* cells after exposure to this peptide is not an uncommon phenotype [71], and this fact led to the necessity of combining it with other hurdles, represented by either antimicrobial substances or physical treatments. Wongchai et al. showed that nisin (62.5 µg/mL) combined with salts of organic acids could overcome this problem, as the synergism with citric acid (1000 µg/mL) prevented the growth of *L. monocytogenes* on pork ham during storage at 4 °C for 4 days [72]. Hammou et al. also noticed that nisin (200 µg/g) combined with NaCl (salt; 12%) can significantly inhibit the growth of the pathogen on natural sheep casing during 90 days of storage at 6 °C [73].

Other researchers focused on the synergism between nisin and EOs as preventive strategy regarding *L. monocytogenes* proliferation in meat and meat products. Raeisi and colleague [74] investigated the fate of *L. monocytogenes* (at an initial concentration of 3.2 log CFU/g) during storage at 4 °C for 15 days on chicken meat coated with sodium alginate that contained either nisin (N) alone or in combination with *Cinnamomum zeylanicum* EO (CEO + N) and rosemary EO (REO + N). The results of the study indicated a better efficiency in controlling *L. monocytogenes* growth of the coatings supplemented with CEO + N (final concentration of 6.4 log CFU/g) and REO + N (final concentration of 6.6 log CFU/g) than that containing only N (final concentration of 7.5 log CFU/g) [74]. Carvacrol, the main constituent of thyme or oregano EOs, was shown to affect *L. monocytogenes* cells by inducing irreversible damages to their cell wall and cellular membrane [75]. Therefore, it is considered a good candidate in combating the occurrence of *L. monocytogenes* resistance against nisin. Indeed, the pathogen’s growth on sliced bologna sausage was significantly decreased in samples treated with nisin (25 µg/mL) together with carvacrol (62.5 µg/mL) compared to those treated with these antimicrobial substances separately. Moreover, due to the synergism between the two additives and, as such, the side effects concerning the sensorial properties, consumers’ acceptance towards meat products treated with EOs can be increased [75].

Nisin was shown to increase the *L. monocytogenes* inactivation rate in meat products by high pressure processing (HPP) [76,77]. Teixeira et al. [78] obtained a reduction in *L. monocytogenes* counts on ham by more than 5 log CFU/g, when the meat product was subjected to a combined treatment consisting of HPP (500 MPa, 5 °C, 3 min) and nisin (~2 µg/cm^2^). Moreover, after 4 weeks of refrigerated storage of ham, *L. monocytogenes* cells remained undetectable [78]. By achieving microbial safety, synergism with nisin may also contribute to the reduction in the HPP expenses at a level comparable to that of the traditional methods of meat products’ processing, such as heat treatments [76]. This bacteriocin was also successful in the enhancement of gamma radiation treatments against *L. monocytogenes* [79,80,81]. The study conducted by Mohamed and co-workers combined gamma radiation with nisin and showed an antimicrobial additive effect against *L. monocytogenes*, during the first 24 h after treatment, and a synergistic one, during the next 48 h of storage at 4 °C. The authors suggested as a potential strategy for *L. monocytogenes* elimination the combined treatment consisting of nisin (10^3^ IU/g) and gamma radiation applied at 1.5 kGy [82].

The next most studied bacteriocin in *L. monocytogenes* biocontrol research is pediocin. The pediocin-like peptides belong to class IIa of bacteriocins, being produced by *Pediococcus* spp. and described as biologically active against *Listeria* spp. [83,84]. Their effectiveness in the reduction in *L. monocytogenes* load in meat products by direct addition or produced by LAB strains has been demonstrated by former studies [85,86,87]. More recently, the biocontrol of *L. monocytogenes* by pediocin was evaluated by its incorporation in active packaging materials. Such materials are intended to inhibit or delay the growth of undesired microorganisms while minimizing preservatives’ addition to food products [88]. The in vivo approach showed encouraging results [89]. For instance, Woraprayote [90] developed an antilisterial poly lactic acid/sawdust particle biocomposite film incorporated with pediocin PA-1/AcH. The highest anti-listeria activity was achieved by pediocin adsorption to the coating at 11.63 ± 3.07 μg protein/cm^2^. The authors indicated that the obtained material’s potential of *L. monocytogenes* inhibition in contact with the contaminated raw sliced pork could be of 99%, as suggested by a model study [90]. Another study assessed the efficacy of a film based on cellulose containing 25% or 50% pediocin against *Listeria* spp. on sliced ham. While the film containing 25% pediocin could not prevent the growth of *L. innocua*, the one containing 50% pediocin reduced the bacterium by 2 log cycles after 15 days of storage at an abusive temperature (12 ± 1 °C) compared to the control (without bacteriocins) [88]. Pediocin was shown to enhance the inactivation of *Listeria* in meat products treated with HPP. The HPP (300 MPa, 10 °C, 5 min) in conjunction with ex or in situ pediocin bacHA-6111-2 production was applied in the study of Castro et al. [91] to inactivate *L. innocua* inoculated in fermented meat sausages and to evaluate the survival of the bacterium during 60 days of storage at 4 °C. Considering a contamination level more likely to occur during the sausages’ processing (~10^4^ CFU/g), it was shown that both ways of pediocin production resulted in a synergistic effect with HPP, as the counts of *Listeria* spp. after the combined treatment decreased by >2 log CFU/g. However, the analysis of bacterium behavior during the storage of sausages revealed that in situ bacteriocin production was more efficient regarding the control of its growth [91].

### 3.5. Essential Oils in Meat Products

Many studies evaluated EOs’ potential to control *L. monocytogenes* in meat and meat products. The aromatic oils have been applied either as such [81,92,93,94], encapsulated [95], or incorporated into edible coatings [96] (Table 2). The last two delivery systems were reported to be more acceptable to consumers in terms of meat products’ organoleptic properties after being applied. Besides this, the encapsulation of EOs and their addition to various active packaging materials have been shown to solve the inconveniences regarding EOs’ instability to external factors [43] and poor solubility in foods with low fat content [36]. Also, due to the need of relatively high amounts of EOs to achieve a satisfactory degree of pathogens’ inactivation, the treated meat products can become inappropriate for consumption as a result of altered sensorial characteristics and possible toxicity [97]. Lower concentrations of EOs can be used if combining them with other natural antimicrobial substances, such as bacteriocins [98] or physical treatments, results in a synergistic effect. Bearing in mind that minced beef supplemented with 0.9% thyme oil was unacceptable in terms of organoleptic properties, to achieve a sufficient inactivation degree of *L. monocytogenes*, Solomakos et al. instead recommended a combined treatment consisting of 0.6% thyme oil and 1000 IU/g nisin [98]. In the study by Huq et al. [81], it was shown that during storage at 4 °C, the synergism between oregano (250 µg/mL) or cinnamon (250 µg/mL) EOs and nisin (16 µg/mL) determined slower growth rates of *L. monocytogenes* on cooked ham (0.20 and 0.11 ln CFU/g/day, respectively) compared to EOs alone (0.21 and 0.18 ln CFU/g/day, respectively) [81]. Moreover, the encapsulation of cinnamon EO combined with nisin and the treatment of the RTE ham with the obtained capsule resulted in a much lower growth rate of the bacterium, of 0.05 ln CFU/g/day. The increased efficiency was attributed to a better preservation of the antimicrobials’ biological activity when entrapped in the biopolymeric matrix and their better distribution on the meat product’s surface [81,99]. 

The nanoemulsion of EOs has been shown to generate better results in terms of *L. monocytogenes* biocontrol in comparison with emulsions. Some of the advantages of using EOs in this form are the improved stability and increased physical resistance of the EOs, and better transferability of the hydrophobic bioactive compounds. Kazemeini [100] compared the antimicrobial activity of an alginate edible coating containing the nanoemulsion of *Trachyspermum ammi* EO to that of the coating containing the emulsion of the same EO against *L. monocytogenes* inoculated on turkey fillets. By the end of the contaminated meat product’s storage (4 ± 1 °C for 12 days), the counts of *L. monocytogenes* were lower in the meat treated with *T. ammi* EO nanoemulsion (7.12 ± 0.09 log CFU/g) compared to that treated with *T. ammi* emulsion (5.53 ± 0.13 log CFU/g) [100].

Dini et al. [101] assessed the efficacy of a chitosan film containing 1% nanoemulsion of cumin EO combined with low-dose gamma irradiation (2.5 kGy) against *L. monocytogenes* on beef loan during refrigerated storage. While the edible film alone did not exert a good control on the pathogenic bacterium’s growth, its combination with the physical treatment generated an enhanced antilisterial effect, which might become an effective strategy to ensure the microbiological safety and improved shelf-life of the meat product [101]. Khaleque et al. [94] showed that the reduction in *L. monocytogenes* population in ground beef treated with 5% clove EO was more accelerated at refrigeration (8 °C) and chill (0 °C) temperatures compared to the storage at freezing temperatures (−18 °C) [94]. Solomakos and colleagues noticed that the antilisterial effect of EOs was also influenced by the storage temperature, and a stronger antimicrobial activity of thyme EO against the pathogen in minced beef was found when stored at 10 °C than at 4 °C [98].

**Table 2:** Studies exploring the application of essential oils as biocontrol tools against *L. monocytogenes* in meat and meat products.

Meat or Meat Products	Application of EOs	Contamination Procedure	Storage Conditions and Results	References
Beef meatballs	Addition of *O. vulgare*, *R. officinalis,* and *T. vulgaris* at concentrations of 0.5%, 1%, or 2% (*v*/*w*)	Inoculation with a five-strain cocktail *(L. monocytogenes* HSD 2434, HSD3261, HSD 3705, HSD 3948, HSD 4210) at 10, 10^2^, 10^3^, and 10^4^ CFU/g	Concentrations of 2% and 1% restricted the growth of *L. monocytogenes*, regardless of the initial microbial loading, during storage at 4 °C for 14 days, but affected the meatballs flavor. Concentration of 0.5% restricted the growth of *L. monocytogenes* at initial counts of <10^2^, and the taste of meatballs was acceptable.	[92]
Italian mortadella	Addition of combined *T. vulgaris* and *R. officinalis* at concentrations of 0.025% and 0.05% during manufacturing	Contamination of mortadella slices with a three-strain cocktail (*L. monocytogenes* ATCC 19111, ATCC 13932, and ATCC 19117) at ~2.5 log CFU/g	Compared to the untreated contaminated mortadella, addition of combined EOs to the concentrations of 0.025% and 0.05% led to a reduction in *L. monocytogenes* by 2.29 log CFU/g and 2.79 log CFU/g by the end of storage at 4 °C for 30 days.	[102]
Ground beef	Addition of crude and commercial *C. cassia* and *S*. *aromaticum* EOs at concentrations of 5% and 10%, and 2.5% and 5%, respectively	Inoculation with a five-strain cocktail (*L. monocytogenes* ATCC 43256, ATCC 49594, JCM 7676, JCM 7672, and JCM 7671)	The ground beef was stored at 8 °C and 0 °C for 7 days and at −18 °C for 60 days. A 10% concentration of clove EO (both crude and commercial) completely inactivated *L. monocytogenes* within 3 days of storage, irrespective of temperature. A 5% concentration of clove EO (both crude and commercial) reduced *L. monocytogenes* gradually throughout storage, irrespective of temperature, without achieving complete inactivation. The 2.5% and 5% concentrations of crude and commercial cinnamon EO did not inactivate *L. monocytogenes* throughout storage. Consumers did not find the ground beef treated with 10% clove EO acceptable, while some of them found the meat treated with 5% clove EO acceptable.	[60,74,94]
Dry-cured ham-based medium	Addition of *C. cassia* EO in dry-cured ham-based medium with water activity of 0.93 or 0.95 at a concentration of 10%	Inoculation with a serotype 4 *L. monocytogenes* strain at ~4 log CFU/mL	During storage at 7 °C for 7 days, 10% cinnamon EO completely inhibited *L. monocytogenes* growth irrespective of the ham-based medium’s a_w_.	[60]
Fresh chicken meat	Corn starch edible coating containing *Zataria multiflora* EO nanoemulsion alone and fortified with cinnamaldehyde	Contamination of the meat with *L. monocytogenes* to a final concentration of ~10^4^ CFU/g followed by its immersion in the corn starch solutions	The coating with fortified nanoemulsion was more effective in controlling *L. monocytogenes* than that with the nanoemulsion alone during storage at 4 ± 1 °C for 20 days, with a growth difference between the treatments of ~1 log CFU/g.	[96]
Fresh beef	Soy protein edible coatings containing 1%, 2%, or 3% thyme or oregano EOs	Contamination with *L. monocytogenes* at 5.59 log CFU/g followed by beef pieces immersion in the coating solutions	At the end of storage (14 days at 4 °C) period, compared to the uncoated beef pieces, coating with 1, 2, and 3% thyme and oregano EOs reduced *L. monocytogenes* by 1.02, 1.73, and 1.97 log CFU/g and 0.91, 1.66, and 1.90 log CFU/g, respectively. The treatments improved the color of beef, and its organoleptic properties were acceptable.	[103]
Spiced beef	Chitosan films incorporated with apricot (*Prunus armeniaca*) kernel EO at 0%, 0.125%, 0.25%, 0.5%, and 1% (*v*/*v*)	The beef slices were inoculated with *L. monocytogenes* to 10^4^ CFU/g and placed in contact with the antimicrobial films	After 15 days of storage at 4 °C, compared to the control samples (film without EO addition), the chitosan films containing 0.5 and 1% apricot kernel EO reduced *L. monocytogenes* by 3.3 and 4.1 log CFU/g. After 24 days of storage, the sensorial attributes (taste, color, texture, and overall acceptance) of the spiced beef packed with the chitosan film containing 1% apricot kernel oil were significantly improved compared to those of the unpacked one.	[104]

## 4. Biocontrol of *L. monocytogenes* in Milk and Dairy Products

One of the challenges of controlling *L. monocytogenes* in dairy products is that this bacterium can form biofilms on equipment, pipes, or other specific utensils, being considered a source of contamination if we take into account that these bacteria are able to detach from the biofilm and contaminate the products on different steps of production. According to criteria established by the European Union (EC 2073/2005) regarding RTE soft cheese products as well as other RTE products that may permit *L. monocytogenes* growth, the value of 100 CFU/g is the limit set up for their shelf-life [105]. The main soft cheese properties that facilitate the survival of *L. monocytogenes* refer to pH values over 6.0, the maximum NaCl content of 3.5% (*w*/*w*), and the water activity (aw) value above 0.94, together with its capacity to grow in refrigeration conditions. Although *L. monocytogenes* is inactivated by thermal treatments, in cheese processing plants, the contamination may occur in the ripening process upon brine solution addition or after pasteurization, due to the poor hygiene design manufacturing as the main reason or as a result of resistance to sanitizers and disinfectants [106,107,108,109].

### 4.1. Bacteriophages in Milk and Dairy Products

Several studies have been published to evaluate the effectiveness of phage-based inhibition of *L. monocytogenes* in dairy products, such as raw milk, pasteurized milk, cheese, yogurt, butter, and cream.

Phage efficacy is largely influenced by the food matrix structure, according to Guenther et al. [110]. The dairy products have complex matrices with microstructures that vary depending on how the milk is processed and stored, which may affect how phage and bacterial cells interact [111]. 

The commercial solution ListShield (Intralytix, Baltimore, MD, USA) was applied to the surface of a cheese matrix (with varying pH levels), at a concentration of 8 × 10^6^ PFU/g, in order to evaluate the growth of *L. monocytogenes* (serotypes 1/2a, 1/2b, and 4b) at different temperatures (6 °C, 14 °C, 22 °C), for a period of 14 days, in a laboratory-scale model [112]. The results obtained emphasized that the effectiveness of the cheese phage treatment depends on the pH, temperature, and *L. monocytogenes’* serotypes. More precisely, the phage cocktail could reduce by ~1 log the *L. monocytogenes* counts, on the first days of storage at the highest temperature that was tested and a pH of 6.5 [112]. The most important safety aspect that arises from a number of studies refers to the regrowth of *L. monocytogenes* on cheese treated with phage, during long-term storage even with an initial *Listeria* count reduction [53,113,114,115,116]. In another study, the effectiveness of Listex P100 was determined in reducing *L. monocytogenes* on both pasteurized milk and broth media as a function of storage temperature and duration. The study emphasized that Listex P100 reduced the bacterial counts by 1.5 to 3.5 log CFU/mL in milk and by 2.5 to 4.5 log CFU/mL in broth media, in correlation with the storage conditions [115]. In addition, the moment of bacteriophage addition in the experiment set-up (before or after *L. monocytogenes* inoculation) represents a requirement for the total elimination of the pathogenic bacteria. 

In a systematic review, Romero-Calle et al. [116] evaluated using a meta-analysis of the efficiency of patented phages as the biological control for two foodborne pathogens, including *Listeria* spp. and *Salmonella* spp. The results highlighted that ListShield™ and Felix01 phages were the most effective in the reduction in *Listeria* spp. from different food matrices even of dairy products [116].

The combined effect of two *Listeria* phages, FWLLm1 and FWLLm3 (*Myoviruses* isolated from sheep feces), and the coagulin C23 as bacteriocin were tested by Rodríguez-Rubio et al. [117] aiming to eliminate the *L. monocytogenes* in milk under refrigeration conditions. The combination FWLLm3 + C23 was more effective than FWLLm1 + C23 in lysing the *L. monocytogenes* cells when the same ratio of phage-to-bacteria was used. The former combination reduced the bacterial counts below the detection limits after 2 h, while the latter combination allowed the re-growth of *Listeria* cells [117]. In a recent article, Elsayed et al. [118] isolated and characterized six different phages from dairy cattle environments and assessed their antimicrobial effect against 22 different multidrug-resistant *L. monocytogenes* strains alone and in conjugation with silver nanoparticles (AgNPs). The authors showed that the bacteriophages attached to silver nanoparticles were protected from the harsh environmental conditions, enhancing their effectiveness in reducing *L. monocytogenes* growth (without regrowth) and excluding the possibility of phage to induce bacterial resistance. 

The published results were generally positive, showing that phages can reduce or eliminate *L. monocytogenes* in dairy products, either alone or in combination with other factors, such as low pH, salt, bacteriocins, or AgNPs. However, there are also some challenges and limitations for the application of phages in the dairy industry regarding the variability of dairy products and processing conditions, which can affect the stability and activity of phages, and the possibility of development of *L. monocytogenes* phage-resistant mutants, which can emerge and compromise the efficacy of phage treatment. Moreover, the lack of standardization and regulation for the production, quality, and safety of phage products can hinder their commercialization and acceptance by consumers and authorities. However, more research and development are needed to optimize the phage application methods, to monitor the phage and bacterial dynamics, and to establish the regulatory and legal frameworks for the use of phages in the dairy industry. 

### 4.2. Lactic Acid Bacteria and Bacteriocins in Dairy Products

By increasing the requirements for products with functional properties derived from fermentation, new probiotic LAB strains have been isolated and screened for their antilisterial properties in microscale cheeses approach, along ripening. The *Lpb. plantarum* (1QB77) strain highlighted the potential to reduce *L. monocytogenes* with 2.5 log CFU/g, at the end of the ripening experiment (21st day) [119,120]. 

Martín et al. [70] suggested that LAB-producing anti-*L. monocytogenes* peptides (bacteriocins) or the LAB strains alone could be a promising tool to control this pathogen in dairy-ripened products. To prove the antilisterial activity of LAB strains in a “Torta del Casar” cheese-based agar model system, following industry storage conditions (7 days at 7 °C) [70], created 20 different combinations of which those between *Lactiplantibacillus plantarum* (B2) and *Lactiplantibacillus* spp. (B4) were the most effective, the reduction being higher than two logarithmic units [70]. 

The effect of lactolisterin BU, the bacteriocin produced by *Lactococcus lactis* subsp. *lactis* BGBU1-4, was tested, on the *L. monocytogenes* ATCC19111 strain, artificially inoculated in the Quark-type cheese, during 21 days of storage at 4 °C. The adjunct culture BGBU1-4 highlighted excellent antilisterial activities (with the reduction of 2.6 log cfu/g), being considered as a potential bacteriocin-producing biocontrol strain [121]. Kondrotiene and co-workers [122] reported a reduction in *L. monocytogenes* of 2 log units within 7 days of cheese storage, by using three nisin-producing *Lc. lactis* strains. By directly inoculating the milk with bacteriocinogenic *E. faecalis* strains for fresh cheese production, a higher *L. monocytogenes* count reduction of 3–4 log units was achieved [123]. 

Recent applications highlighted the advantages of using bacteriocin-producing LAB strains [123,124,125] over the use of only bacteriocins, considering that the bacteriocins are easily degraded into cheese matrices. To counteract this effect and to better control *L. monocytogenes*, some bacteriocins were incorporated into fresh cheese packaging films (enterocin from *E. avium* DSMZ17511 on agar edible films, achieving a *L. monocytogenes* reduction of 1 log unit [126], enterocin whey solution from *Enterococcus faecalis* L2B21K3 and L3A21K6 into gelatin/glycerol films, having a reduction of 5 log CFU/g from 5th to the 30th day of the experiment [127], or bacteriocins of *Pediococcus pentosaceus* 147 into chitosan-based edible coating [128]. Moreover, the immobilization of entire cells of *Lactococcus lactis* L3A21M1 and *Lc. garvieae* SJM17 into an edible fresh-cheese coating containing alginate, maltodextrin, and glycerol significantly reduced the *L. monocytogenes* and prevented the pathogen migration into the cheeses [129]. 

The combination of selected LAB strains with different antimicrobial compounds specially bacteriocins, followed by their immobilization into edible films to create an active packaging, could be the next future innovation for eliminating the risk of *L. monocytogenes* in dairy products.

### 4.3. Essential Oils in Dairy Products

Natural preservatives such as essential oils and plant extracts were added to cheese due to their strong antimicrobial properties against cheese pathogens and spoilage microorganisms, respectively. Besides affecting the activity of lactic acid bacteria or the products’ sensory attributes, the interaction of essential oils with fat, carbohydrates, or proteins in cheese may reduce their antimicrobial effectiveness since an increased amount of EOs is necessary to maintain the antimicrobial activity and the safety of the products. Gouvea and co-workers [130] published a review in which they highlighted the impact of essential oils and plant extracts on the microbial and sensory aspects of different cheese products. 

In a study by Da Silva Dannenberg et al. [131], conducted over a period of 30 days of storage under refrigerated conditions, it was shown that *L. monocytogenes* growth kinetic varied depending on the concentration of the EOs added and also by its provenance. The fresh cheese treated with 2% of EO from the mature pink pepper fruits had the highest *L. monocytogenes* (2 × 10^4^ CFU/g) inhibitory activity from all the treatments, at the end of the experiment [131]. 

The antibacterial effect of carvacrol, clove, and cumin EOs, as well as their nano-emulsions (NEs), were tested on *L. monocytogenes* inoculated on Egyptian Talaga cheese, made in the lab, and stored in the fridge. When added to cheese, NEs significantly lowered the number of pathogen cells from 8.2 log10 CFU/g to 1.5 log10 CFU/g after 2 to 3 weeks, while EOs took 4 to 5 weeks to have the same effects. The carvacrol NE had the best antibacterial activity and did not affect the cheese sensory quality. The reduction in *L. monocytogenes* cells by carvacrol NE was 99% after 7 days at the concentration of 0.78%, while NEs of the other EOs needed higher concentrations and longer time to show the same effect [132].

### 4.4. Endolysins in Different Antilisterial Formulae of Dairy Products

The efficacity of endolysin PlyP100 (10 U/g) towards a *L. monocytogenes* serotype cocktail (6a, 4b, 1/2a, 1/2b) was assessed in Queso Fresco (QF—a Hispanic-style fresh cheese), in a combination formula with nisin (250 µg/g) [133]. After 28 days of storage at 4 °C with antimicrobial mixture treatment, the *L. monocytogenes*’ concentration was reduced by 4 log CFU/g, in approximately half of the tested QF samples. None of the individual treatments were enough to eradicate the pathogen either in QF matrices or milk [133,134].

On the other hand, the nisin A treatment was not very effective in some foods, especially in QF cheese, due to its high fat content (>20%) and neutral pH. In this regard, Ibarra-Sanchez et al. modified the metabolic pathway of *L. lactis* strain to enable the synthesis of nisin A derivatives by replacing two hydrophobic residues (I30 and V32) with positively charged amino acids (H, K, and R). Of all the nisin derivatives, the H27/31K was more stable and had an increased antilisterial activity compared to nisin A, when it was tested in QF matrices. The combined formula of H27/31K with endolysin PlyP100 proved to be faster and more effective in eliminating the *L. monocytogenes* in QF than nisin A + PlyP100, a significant reduction of 3.5 log CFU/g being achieved after 28 days of storage under refrigeration conditions [135].

## 5. Biocontrol of *L. monocytogenes* in Vegetables and Fruits

### 5.1. Bacteriophages in Vegetables and Fruits

Leafy vegetables (spinach, lettuce, and rocket) are prone to contamination with harmful germs such as *L. monocytogenes* especially due to their contact with soil or water sources, where this bacterium lives as a saprophyte [136]. There were two recent multistate outbreaks in the United States linked to the consumption of leafy greens. Both of them officially ended in 2022 and involved packaged salad and led to multiple hospitalization cases and deaths [137]. 

As it has been specified before, there are several commercial phage products developed for the control of *Listeria* spp. in foods, such as PhageGuard Listex, ListShield™, and Listex™ P100 [54,138]. Their use as surface treatment for leafy vegetables has been shown to be effective in terms of *L. monocytogenes* reduction immediately after application. PhageGuard Listex applied on curly endive through a spraying system at two places on the processing lines, conveyer and centrifuge, respectively, reduced the concentration of listerial cells by 2.5 log CFU/g. Three days after the treatment, the concentration of *L. monocytogenes* on endive decreased even more, by 3.5 log CFU/g [139]. Also, the treatment with ListShield™ phage product, a cocktail of six lytic bacteriophages, at a concentration of 10^8^ PFU/mL for 10 min reduced significantly the *L. monocytogenes* cells within biofilm formed on Romaine lettuce (up to 0.75 log CFU/cm^2^) [138]. The same product reduced nalidixic acid-resistant *L. monocytogenes* on fresh spinach placed under either unmodified or modified atmosphere in sealed packages. After 14 days of storage at 4 and 10 °C (abusive temperature), the *L. monocytogenes* population on spinach leaves was lowered by 1.51 and 2.51 log CFU/cm^2^, respectively, in the case of atmospheric air packaging, and by 1.95 and 3.24 log CFU/cm^2^, respectively, in the case of modified atmosphere packaging [140]. 

Phage treatment could also prevent the growth of *L. monocytogenes* in fresh-cut fruits and fruit juices. The treatment with Listex P100 (10^8^ PFU/mL) showed high efficiency in melon slices and juice, where the *L. monocytogenes* population decreased by 1.5 log CFU/plug and 8 log units, respectively, after 8 days of storage at 10 °C. In more acidic fruits, such as pears, the treatment was less effective (reduction of 1 log CFU/plug and 2.1 log CFU/mL for slices and juice, respectively) or ineffective in the case of apples [141]. 

The isolation of new anti-*Listeria* bacteriophages, in order to improve the efficiency of this biocontrol method, is an ongoing research effort. For instance, three lytic bacteriophages were successfully isolated against *L. monocytogenes* entitled LMPC01, LMPC02, and LMPC03 from sewage, river, and soil, respectively. When combined, the three phages reduced the bacterium on celery and enoki mushrooms by 2.2 and 1.8 log CFU/g, respectively, during storage at 4 °C for 7 days [142,143]. 

Current research suggests that the efficacy of bacteriophages regarding *L. monocytogenes* cell reduction on foods depends on a series of factors: food matrix properties, storage temperature, the stage of bacteria growth, and multiplicity of infection (MOI) [142]. Stone et al. [142] observed that after the treatment of baby spinach with phage vB_LmoH_P61, at a concentration of ~2 × 10^8^ PFU/g, the reduction rate of the pathogenic bacterium differed depending on the temperature at which the vegetable was stored. After 6 days of storage at 8, 12, and 25 °C, the concentration of *L. monocytogenes* decreased by 1.93, 2.06, and 3.3 log CFU/g, respectively. An explanation of these results might be the faster growth of the host cells at 25 °C compared to the other two storage temperatures, which increases the chance of bacteriophages to encounter the listerial cells. 

The combination of lytic phages with antimicrobial substances could enhance the reduction in *L. monocytogenes* on vegetables. Oladunjoye et al. [144] noticed an improved lysis capacity of the phages (Listex P100; 10^8^ PFU/mL), when tomato and carrot wedges were concomitantly treated with sucrose monolaurate at 400 ppm. The same authors showed that Listex P100 phage (10^8^ PFU/g) product in combination with trisodium phosphate (TSP) could also represent an effective biocontrol tool against *L. monocytogenes* in fresh-cut produce. Although, at 10 mg/mL, the phage-TSP treatment functioned only in the case of fresh-cut melon, the increase in the TSP concentration at 30 and 60 mg/mL resulted in *L. monocytogenes* reduction on fresh-cut tomato by 1 and 2 log CFU/mL and by 2 and 5 log CFU/mL during storage at 4 °C and 10 °C, respectively, for 6 days [144].

### 5.2. Lactic Acid Bacteria and Bacteriocins in Vegetables and Fruits

The results of several studies support the potential of LAB as an effective biocontrol method of *L. monocytogenes* in vegetables. The co-culture of *L. monocytogenes* with LAB isolated from kimchi on lettuce for 24 h resulted in a reduction in the pathogen by 1.62 log CFU/cm^2^ [145]. Also, the addition of *Pediococcus pentosaceus* DT016 as protective culture in fresh iceberg lettuce, rocket salad, spinach leaves, and parsley decreased the *L. monocytogenes* population by at least 1.4 log CFU/g compared to untreated vegetables [146].

Amado et al. [147] reported that bacteriocinogenic LAB can be used as inoculants to control *L. monocytogenes* during the ensilage of grass and maize. They showed that the addition of *Lactiplantibacillus plantarum* strain CECT 220 alone or in combination with *L. lactis* CECT 539 or/and *P. acidilactici* NRRL B-5627 to these forages resulted in adverse conditions (low pH and anaerobiosis) that prevented the pathogen’s proliferation [147]. Many studies reported the efficacy of this bacteriocin alone or combined with other hurdles regarding *L. monocytogenes* growth inhibition in vegetables and vegetable-based products. The antimicrobial agent was shown to substantially reduce *L. monocytogenes* on fresh-cut iceberg lettuce when applied concomitantly with modified atmosphere packaging, without altering the sensorial properties of the product during storage [148]. Some researchers studied the effect of nisin together with other bio-preservatives, such as EOs, with respect to *L. monocytogenes* control. It was reported that the usage of nisin combined with food-grade oil components (thymol and eugenol) results in a greater degree of inhibition of *L. monocytogenes* biofilm formation on lettuce surface than the treatments applied separately [149]. Good results were also obtained when nisin was added at half the minimum inhibitory concentration (MIC) in combination with carvacrol at MIC/16 [150]. Ndoti-Nembe et al. [151] demonstrated that treatment with nisin (10^3^ IU/mL) and carvacrol or mountain savory EO (0.35% *w*/*w*) combined with low-dose irradiation (1 kGy) could effectively eradicate *L. monocytogenes* on mini carrots. Hence, it seems that the synergism between nisin and EOs allows the addition of these antimicrobials in lower doses than the concentrations required separately for the bacterium inhibition, without compromising foods’ safety and retaining, at the same time, their quality. 

Combining nisin with the salts of organic acids is another approach that was shown to offer a good protection against *L. monocytogenes* in fresh produce. Oladunjoye and colleagues [152] assessed the synergy between nisin and sodium citrate or sodium acetate in terms of pathogen control on tomato slices. In this case, nisin combined with sodium citrate at the concentration of 5% resulted in the highest log reduction in *L. monocytogenes* (~2.27 to 2.28 log CFU/mL), leading to the conclusion that such a treatment can be employed as an efficient method of decontamination with minimum impact on vegetable quality [152].

Although not yet approved as commercial food additives, class II bacteriocins are intensively tested for food preservation applications, with promising results regarding *L. monocytogenes* proliferation prevention in vegetables. From among these, enterocin AS-48 proved to be a very efficient anti-*Listeria* agent. The studies that addressed this topic regarding vegetable products’ bio-preservation (vegetable-based salads, fruits, and fruit juices) applied it alone or in combination with natural (e.g., EOs, phenolic compounds, citric and lactic acid, other bacteriocins, chitosan) or synthetic preservatives (e.g., sucrose palmitate, sucrose stearate, trisodium trimetaphosphate) to enhance the pathogen’s inhibition degree throughout storage [153,154,155]. The bacteriocin was also incorporated in edible coatings, with these being used against *L. monocytogenes* contaminating apple cubes. The total inactivation of the pathogen was achieved when enterocin AS-48 was used in conjunction with ethylenediaminetetraacetic acid (EDTA) in coatings based on chitosan, pectin, and xanthan gum [156]. Another study tested plastic bags containing enterocin AS-48 and thymol regarding the control of *L. innocua* in fruit puree. The activated films reduced the bacterium by 2 log cycles after 3 days of product’s storage at 5 °C, while, at the end of storage time (10 days), *L. innocua* could no longer be detected [157].

Treatments with pediocin have also been reported as effective strategies in the fight against *L. monocytogenes*. Washing fresh vegetables with a solution containing pediocin DT016 prevented the proliferation of the pathogen during storage. Moreover, compared to the sodium hypochlorite solution (200 μg/mL) and water, pediocin DT106 solution determined a significantly higher degree of *L. monocytogenes* reduction, by 2.7 and 3.2 log CFU/g, respectively [146].

### 5.3. Essential Oils in Vegetables and Fruits 

In this regard, Tao et al. [158] impregnated cellulose stickers with carvacrol, oregano EO, and cinnamon EO at 262, 360, and 556 μL/L headspace, respectively, and placed them on the lids of recipients containing frozen green peppers artificially contaminated with *L. grayi*, a nonpathogenic surrogate for *L. monocytogenes*, at a concentration of ~3 log CFU/g. After storage for 5 days at room temperature, compared to the untreated samples, in which case the *Listeria* population increased by ~5 log CFU/g, the treatments led to a reduction in the bacterial cells by ~2 log CFU/g. Another study incorporated organic acids and rosemary extract combined with either Asian spice EO or Italian spice EO in biodegradable films. These were inserted into containers with contaminated broccoli which were placed at 4 °C for 12 days. In the case of *L. monocytogenes*, the bioactive films demonstrated a good control capacity during the first 4 days of storage [159].

Another possibility to use EOs in food preservation is to incorporate them in edible coatings that can be applied directly on the food products’ surface. Apart from enhanced antimicrobial activity, the benefits of this strategy envisage the protection of the products against physical deterioration over time and reduction in environmental pollution [160]. 

An edible film based on sodium alginate that contained *Citrus sinensis* EO was shown to reduce the pathogenic population formed of *Salmonella* and *Listeria* on tomatoes during storage at 25 °C for 15 days [161]. Jovanović and co-workers [162] applied a chitosan–gelatin film containing thyme oils on black radishes. The addition of EOs enhanced the antimicrobial activity of the coating. The combination of 1% chitosan and 0.2% thyme oil reduced *L. monocytogenes* on the vegetable by ~2 log CFU/g after 24 h of contact. Other authors assessed the efficacy of edible coatings containing EOs combined with physical treatments against *L. monocytogenes* in vegetable products. Such treatments can make the cellular membrane more permeable to antimicrobial substances, thus increasing the reduction rate of microbial populations. In this respect, Severino and colleagues [163] tested the synergistic effect between a bioactive coating based on modified chitosan that contained 0.5% nanoemulsion of mandarin EO and three non-thermal treatments, namely, γ-irradiation, UV-C, and ozonated water, against *L. innocua* in green beans. Among the combined treatments, the variants with the most promising results (reduction of ~3 log CFU/g after processing) were the treatments that included γ-irradiation and UV-C. Moreover, besides a strong synergistic effect, the treatment including UV-C prevented the loss of firmness and color change in beans during storage. Another study evaluated the capacity of a similar film, formulated with 0.05% nanoemulsion of mandarin EO, in combination with high pressure processing (HPP) or pulsed light (PL) against *L. innocua*. The results indicated the treatment combined with HPP (300 MPa, 5 min) as more efficient compared to the one that included PL (1.2 × 10^5^ J/m2), as the concentration of listerial cells was decreased by ~4 and 2.4 log cycles, respectively. Also, during storage at 4 °C for 14 days, the bacterial population recovered better following the coating–LP treatment. However, in terms of beans aspect, the coating–HPP had a greater negative impact on product’s firmness [164].

### 5.4. Endolysins

Regarding the biocontrol of *L. monocytogenes* in vegetables by the addition of endolysins as antimicrobial agents, the specialized literature is still limited. One study assessed the efficacy of the *Listeria* cell lytic enzyme Ply500 in lettuce decontamination. The main challenge of the experiments was to obtain stable formulations so that the enzyme comes into full contact with the bacterial cells to achieve good efficiency. The vegetable was treated with either free Ply500 enzyme, Ply500 immobilized onto silica nanoparticles (Ply500-SNP conjugates), a biocatalytic polymer film containing Ply500-SNP conjugate, or Ply500 immobilized onto edible crosslinked starch nanoparticles (MBP-Ply500). Among the treatments, the application of free Ply500 and Ply500-SNP conjugates led to the complete inactivation of the bacterium, a less striking effect being noticed in the case of MPB-Ply500. The bioactive film was also capable of preventing *Listeria* cells from recovery during the storage of the lettuce at 4 °C [165]. Endolysins were also shown to be effective against *L. monocytogenes* in liquid food matrices based on vegetables. Zhang et al. [15] isolated and purified the LysZ5 endolysin from the genome of *L. monocytogenes* phage FWLLm3. The protein’s capacity of lysis was tested against *L. monocytogenes* in soya milk at 4 °C. After 3 h of incubation, at the concentration of 40 IU/mL, the enzyme reduced the listerial cells to an undetectable level.

## 6. SWOT Analysis for Using Biological Antilisterial Agents in Food Product Formulations

Table 3 presents an analysis that highlights the strengths, weaknesses, opportunities, and threats (SWOT) related to the use of antilisterial agents presented in the manuscript. All of these have to be taken into account by food technologists when deciding to use one or a combination of them in a specific product in order to maximize their benefits.

## 7. Conclusions and Remarks for Future Research

This review provides an update of the methods that food technologists may use for the biocontrol of *L. monocytogenes* in foods. Commercial products are already available for some of the antilisterial agents, making things easier to implement in practice. Combining different antilisterial agents is another possibility that food technologist may successfully use at industrial level, allowing a better adaptation to the food matrix, which varies a lot among food types. The variety of combinations allows the development of synergies, and the effectiveness also increases. When selecting an antilisterial agent, it is also important to consider the factors that affect its action and be fully successful in preventing the growth and spread of *L. monocytogenes* in food products. 

More research and development are needed to enhance effectiveness, refine application methods, and proactively address potential challenges. Challenges like the recovery of sublethally injured cells, especially in meat products, the presence of competitive microbiota, the reformulation of food products (e.g., decreasing the salt content), the possibility to incorporate the antilisterial agents into packaging materials, and the economic viability of food businesses should be addressed. Another future direction to act for is to incorporate data regarding *L. monocytogenes* behavior in the presence of antilisterial agents into models that are used to describe its survival in different foods. This will allow better predictions for risk assessors. 

With the adoption of biocontrol solutions, the food industry demonstrates a commitment towards complying with the stricter standards that regulatory bodies impose to manage the *L. monocytogenes*-associated public health risks.

## Figures and Tables

**Table 1:** Studies exploring the application of bacteriophages as biocontrol tools against *L. monocytogenes* in meat and meat products.

Meat or Meat Products	Contamination Procedure	Antimicrobial Agent	Treatment Conditions	Storage Conditions and Results	References
Fresh beef	Surface inoculation with *L. monocytogenes* LM-94 at 6.2 log CFU/g	ListShield^TM^	1 × 10^9^ PFU/mL spot inoculation followed by incubation at RT for 2.5 h	Reduction by 2.3 log CFU/g after storage at 4 ± 1 °C for 15 days	[56]
Spanish dry-cured ham	Surface inoculation with *L. monocytogenes* S2 at 10^5^ CFU/cm^2^, 10^4^ CFU/cm^2^, and 10^3^ CFU/cm^2^	ListShield^TM^	10^7^ PFU/cm^2^	Reduction below the detection limit (10 CFU/cm^2^) for lower contamination level (10^4^ CFU/cm^2^ and 10^3^ CFU/cm^2^) and by 3.5 log units for high contamination level (10^5^ CFU/cm^2^) after storage at 4 °C for 14 days Reduction below the detection limit in low contaminated samples (10^3^ CFU/cm^2^) after storage at 12 °C for 8 days	[54]
		Listex^TM^	10^9^ PFU/cm^2^	Reduction below the detection limit (10 CFU/cm^2^) for all contamination levels after storage at 4 and 12 °C for 24 h	
Fermented meat sausage (*Alheira*)	Contamination with *L. monocytogenes* Scott A and *L. monocytogenes* 1942 at 10^5^ CFU/g	Listex^TM^ P100	10^8^ PFU/g	Reduction below the detection limit of both strains after storage at 4 °C for 14 days	[57]
Cooked turkey and roast beef	Surface contamination with a four-strain cocktail (*L. monocytogenes* 08-5578, Li0512, Li0529, and ATCC19115) at 10^3^ CFU/cm^2^	Listex^TM^ P100	10^7^ PFU/cm^2^	Reduction by 2.1 log_10_ CFU/cm^2^ and 1.7 log_10_ CFU/cm^2^ in cooked turkey and roast beef, respectively, compared to the control (non-treated samples) during storage at 4 °C for 28 days	[58]
RTE pork ham	Surface contamination with a two-strain cocktail (*L. monocytogenes* B7, AL48/15, and *L. monocytogenes* Scott A) at ~2.5 log CFU/g	Listex^TM^ P100	5 × 10^5^ PFU/g	Reduction to undetectable level after storage at 6–8 °C for 72 h	[59,60,61]

PFU—plaque-forming units; CFU—colony-forming units; and RT—refrigeration temperature.

**Table 3:** SWOT analysis for using bacteriophages, LAB, endolysins, and bacteriocins as antilisterial agents in food.

**Strengths**	**Opportunities**
Are effective if used at the right concentrations. Are generally regarded as safe. Except EOs, they do not impact the taste, texture, and nutritional quality of the food. Are highly specific. Are cost-effective. Are easy to be controlled. Have higher acceptance than other methods. Enhance microbial safety in the food industry. Some LABs are probiotics too. EOs have both antimicrobial and antioxidant activities. Applicable to various food products.	Pathogen-free meals without synthetic additives are in demand. Specialists are looking for alternatives to antibiotics since antimicrobial resistance has grown and is now a worldwide concern. Food processors are interested in preventing recalls, legal liabilities, and the loss of consumer trust. Combination of different antilisterial agents is possible. Some antilisterial agents are commercially available, including phage suspensions (e.g., ListShield™). EOs and bacteriocins may be incorporated in packaging materials.
**Weaknesses**	**Threats**
Before being used in foods, the biological antilisterial agents need regulatory approval. EOs impact the taste of food, if high doses are used. Bacteriophages have low tolerance to unfavorable environmental conditions. LAB do not produce bacteriocins, if they experience low-temperature stress. Distribution of antilisterial agents into the food matrix can be a factor that diminishes their effectiveness.	Lysogenic phages could be vehicles for horizontal gene transfer. Some bacteriocins may induce changes in the diversity of intestinal microbiota in different regions of the gastrointestinal tract.

## Data Availability

No new data were created or analyzed in this study. Data sharing is not applicable to this article.
